# Laminar–Turbulent Intermittency in Annular Couette–Poiseuille Flow: Whether a Puff Splits or Not

**DOI:** 10.3390/e22121353

**Published:** 2020-11-30

**Authors:** Hirotaka Morimatsu, Takahiro Tsukahara

**Affiliations:** Department of Mechanical Engineering, Tokyo University of Science, Chiba 278-8510, Japan; 7515117@alumni.tus.ac.jp

**Keywords:** subcritical transition, spatiotemporal intermittency, direct numerical simulation

## Abstract

Direct numerical simulations were carried out with an emphasis on the intermittency and localized turbulence structure occurring within the subcritical transitional regime of a concentric annular Couette–Poiseuille flow. In the annular system, the ratio of the inner to outer cylinder radius is an important geometrical parameter affecting the large-scale nature of the intermittency. We chose a low radius ratio of 0.1 and imposed a constant pressure gradient providing practically zero shear on the inner cylinder such that the base flow was approximated to that of a circular pipe flow. Localized turbulent puffs, that is, axial uni-directional intermittencies similar to those observed in the transitional circular pipe flow, were observed in the annular Couette–Poiseuille flow. Puff splitting events were clearly observed rather far from the global critical Reynolds number, near which given puffs survived without a splitting event throughout the observation period, which was as long as $10^{4}$ outer time units. The characterization as a directed-percolation universal class was also discussed.

## 1. Introduction

The discontinuous reverse transition of wall-bounded turbulence into a laminar flow is a fundamental problem that has been studied for many years, while the laminar-to-turbulent transition is rather smooth, or its critical point is often well predicted by linear stability theory. Subcritical flows in the reverse transition are known to feature two regimes in competition, namely, laminar and turbulent, in which there occurs large-scale intermittency that coexists spatially with a laminar flow. The large-scale nature of localized turbulence often forms a regular pattern once established. The intermittent structure or formation pattern of localized turbulence varies depending on the flow system, and a number of studies have been conducted on canonical flows, such as a circular pipe flow (CPF) and planar flows. In the CPF, a so-called equilibrium turbulent puff, or simply a “puff,” is localized in the streamwise direction, resulting in uni-directional intermittency. The puff turbulence is sustained within a Reynolds-number range based on the bulk velocity *U* and the pipe diameter *D* of $Re_{D}=2000$–2700 [1]: Although there are some differences depending on the experimental conditions, such as the disturbance introduction method and the pipe length [2,3,4], studies have indicated that the puff’s nature is deeply related to the determination of the lower-limit Reynolds number (the global critical Reynolds number, $Re_{g}$), above which turbulent motions can survive globally. Streamwise-localized solutions underlying the puff have been found, and Hopf bifurcations to new branches including unstable periodic orbits are expected to cover the turbulent attractor [5]. It is also known that puffs can split (or proliferate) more frequently than their decay and have a finite lifetime even at $Re>Re_{g}$ [6,7,8,9]. Avila et al. [10] identified $Re_{g}=2040\pm 10$ for the CPF by monitoring both the puff-splitting time and the decay time. Recent attempts have been made to elucidate the puff-driving mechanism [11] and one-dimensional modeling [12], and an understanding of the puff’s nature is progressing compared to other intermittent structures.

In planar flows, intermittent structures with bi-directionality were discovered during the last two decades (excluding the spiral turbulence in a Taylor–Couette flow [13]), which are called oblique turbulent stripes/bands with a certain inclination with respect to the streamwise direction, and were found in a plane Couette flow (pCf) [14,15,16,17,18] and a plane Poiseuille flow (pPf) [19,20,21]. A wall turbulence that is stably stratified by body forces, such as the Coriolis force and buoyancy, also undergoes the stripe regime [22,23]. The stripe pattern has attracted recent interest, and some studies have found families of relevant localized solutions [24,25]. As the Reynolds number approaches the relevant $Re_{g}$, the stripe pattern becomes isolated oblique bands, which fall into a non-equilibrium state accompanied by band growth, a break (not the same as the splitting of the puff), and a mutual collision [26,27,28]. Because the laminar gap surrounding the isolated bands is large at near criticality, a large-scale channel setup or computational domain is required for precise tracking of the process toward a fully laminar state and for estimating $Re_{g}$. For this reason, research is still ongoing, such as elucidating the mechanism of an isolated oblique band [29] and the statistical characteristics [30]. For details, also see recent review papers [31,32,33].

The two kinds of intermittent structures mentioned above were observed in different canonical flows, that is, the CPF and the planar flow, and the direct relationship between the turbulent puff and stripe is unknown. Our research group therefore focused on an annular flow between concentric cylinders. Depending on the radius ratio $\eta \equiv r_{\mathrm{in}}/r_{\mathrm{out}}$ (where $r_{\mathrm{in}}$ and $r_{\mathrm{out}}$ are the inner and outer cylinder radii, respectively), the curvature and the circumferential length (relative to the gap width) should change and may affect the large-scale nature of the intermittency. With η≈1 or 0, the flow system can be regarded as a planar flow or a CPF system, respectively. Ishida et al. [34,35,36] conducted direct numerical simulations (DNSs) to study the subcritical transition process of the annular Poiseuille flow (aPf) using η as a parameter in addition to the Reynolds number. The authors observed both the turbulent puff and the stripe according to η, i.e., a helical turbulence (i.e., a turbulent stripe in the annular flow) at η≥0.5, puff turbulence similar to the transitional CPF at η<0.2, and an intermediate state at 0.2≤η≤0.4. At η=0.1, the observed puff split and decayed over time. A similar tendency was also uncovered in an annular Couette flow (aCf); it was reported that puffs occur at η=0.1, and they split and attenuate over time [37,38]. The authors found a speckled irregular intermittent structure that differs from turbulent stripes and puffs, which was shown to have characteristics of the (1+1)-dimensional directed-percolation (DP) universal class. Recent studies have focused on the relationship between the subcritical transition phenomenon and DP [39,40,41,42].

In this study, by employing an annular system as a platform, we aim to unify uni- and bi-directional intermittent structures observed in the CPF and planar flows, respectively. The key to achieving this aim is bridging between the two different systems in terms of the base flow. The base flows of the studied aPf and aCf are qualitatively different from that of the CPF. This mismatch motivated us to simulate the annular Couette–Poiseuille flow (aCPf) at a low η, which should be more similar to the CPF. However, the presence of the inner cylinder may affect both the onset and splitting of the puff. The main purpose of this study is to answer whether puff splitting would occur in a low η. Moreover, $Re_{g}$ and the Reynolds-number dependence of puff splitting are investigated, and the DP feature is discussed.

Previous DNS studies on Couette–Poiseuille flow mainly focused on the planar turbulence. Kuroda et al. [43] compared the mean velocity profiles and various turbulence statistics for three patterns of imposed mean pressure gradients in the flow path. In particular, among the three patterns, the authors analyzed the shear stress near the moving wall surface in a turbulent field such that it approaches zero. A similar attempt was also conducted by other researchers [44,45,46]. As an experimental study, Nakabayashi et al. [47] also measured the turbulence statistics of a plane Couette–Poiseuille flow at high Reynolds numbers, and classified the flow field into a Couette- or Poiseuille-type depending on the base flow. In addition, Klotz et al. [48,49] eliminated the net flow in a plane Couette–Poiseuille flow (pCPf), allowing localized turbulence to be tracked for long periods of time while stationary in the observation window. A recent quench experiment on the decay of Couette–Poiseuille turbulence is likely to approach the crossover of the decay rate, that is, the quantitative identification of $Re_{g}$ [50]. However, to the best of our knowledge, except for in limited studies [51,52], there is no DNS available for the subcritical transition process of the aCPf. The present DNS is the first to explore laminar–turbulent intermittency in a low-η aCPf.

The remainder of this paper is organized as follows. Section 2 presents the flow configuration, dimensionless parameters, and equations used in our simulations. In Section 3, which is dedicated to the preliminary results, we validated the current code and illustrated the parameter dependence of the base flow in terms of the mean friction on the inner cylinder. Section 4 begins with a puff characterization of the observed turbulent patches. Space-time diagrams of a turbulent quantity revealing the puff splitting and decay are then presented. All results are summarized and discussed in Section 5.

## 2. Problem Setup and Methods

The problem under consideration is the turbulent annular flow of an incompressible Newtonian fluid, for which the governing equations are the equation of continuity and the Navier–Stokes equation, as described in the classical cylindrical coordinate system of (x,r,θ):

(1) $$\begin{array}{ccc} \nabla^{*}\cdot u^{*} & = & 0, \end{array}$$

(2) $$\begin{array}{ccc} \frac{\partial u^{*}}{\partial t^{*}}+\left(u^{*}\cdot \nabla^{*}\right)u^{*} & = & -\nabla^{*}p^{*}+\frac{1}{Re_{w}}\Delta^{*}u^{*}-\frac{dP^{*}}{dx^{*}}e_{x}. \end{array}$$

**Figure 1:**
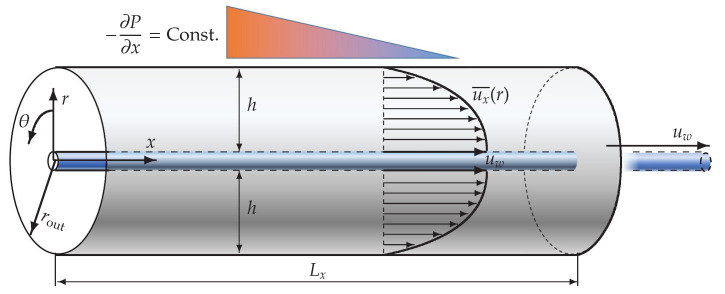
Couette–Poiseuille flow in an annular channel between two concentric cylinders with a radius ratio of $\eta \equiv r_{\mathrm{in}}/r_{\mathrm{out}}=0.1$, driven by a constant pressure gradient and an inner-cylinder axial movement. In this study, the pressure gradient is adjusted such that the mean velocity gradient on the inner-cylinder surface is approximately zero; that is, $\tau_{\mathrm{in}}=\mu {\left[\partial \bar{u_{x}}\left(r\right)/\partial r\right]}_{r=r_{\mathrm{in}}}\approx 0$.

Here, the velocity vector is represented by u, or $\left(u_{x},u_{r},u_{\theta }\right)$, which are the respective components in (x,r,θ); *p* is the pressure, and *t* is the time. These quantities are non-dimensionalized and are marked by a * superscript: $u^{*}=u/u_{w}$, $p^{*}=p/\rho u_{w}^{2}$ (ρ, density), $t^{*}=tu_{w}/h$, $x^{*}=x/h$, and $r^{*}=r/h$, where $u_{w}$ and *h* are the inner-cylinder axial velocity and the gap between the two cylinder radii, respectively, as illustrated in Figure 1. The Reynolds number $Re_{w}$ is therefore based on $u_{w}$, *h*, ρ, and the fluid viscosity μ, whereas another definition using one-quarter of $Re_{w}$ is more conventional for studies on the pCf [15,16,22]. In only the axial-direction component of Equation (2), a constant pressure gradient in *x* is added as an external force term, −dP/dx, with the axial unit vector $e_{x}$. In addition to the imposed pressure gradient, the flow is driven by an axial translation of the inner rod with a constant velocity of $u_{w}>0$. The *x*-axis corresponds to the central axis common to both cylinders, and the radius ratio of η is an important geometrical parameter and is set to 0.1 for the main analysis in this study. Periodic boundary conditions are imposed in both the *x* and θ directions, and no-slip boundary conditions are enforced at the wall surfaces of the cylinders. In the following sections, the imposed pressure gradient is re-defined as the pressure gradient function F(p), which is normalized as

(3) $$F\left(p\right)\equiv -\frac{dP^{*}}{dx^{*}}\cdot Re_{w}=\frac{-dP/d(x/h)}{\mu u_{w}/h}$$

and can be interpreted as the ratio of the imposed pressure gradient (i.e., the Poiseuille-like driving force) against the wall-bounded viscous shear stress (the Couette-like driving force).

As introduced above, there are two control parameters for the flow under consideration, i.e., $Re_{w}$ and F(p). Poiseuille-like flows are realized for a large F(p), whereas Couette-like flows are obtained for a small F(p), and a specifically pure Couette flow corresponds to F(p)=0. As indicated in [44], the ratio of the shear stress at the two walls, which can be defined by $\gamma =\tau_{\mathrm{in}}/\tau_{\mathrm{out}}$ in an aCPf, is another candidate of the control parameter relevant to a Couette–Poiseuille flow. Flows with γ≈0, or a shear-less inner cylinder wall, are of special interest because they exhibit nearly zero mean shear at the moving rod, and can thus be a model for an understanding of the puff dynamics in a pure pipe. Under such conditions, the inner cylinder practically affects the core flow only as an impermeable thin rod, and the coherent turbulent structures and turbulent production that dominantly occur near the static outer cylinder wall mimic those found in a canonical system of the CPF. Although the system chosen here is closer to a CPF than to an aPf or an aCf, it should be noted that the different boundary conditions regarding the inner rod preclude a mathematical homotopy continuation with the CPF. Except for a fully laminar flow state, F(p) providing γ=0 is not explicitly obvious, and thus, a parametric survey must be conducted for each given $Re_{w}$. In this study, we conducted a preliminary survey of the F(p) dependence of $\tau_{\mathrm{in}}$ for several $Re_{w}$ values using a DNS with a medium-scale computational domain, as reported in Section 3. Based on these results, we selected F(p), which will provide $\tau_{\mathrm{in}}\approx 0$ (γ≈0) at each tested $Re_{w}$, and accordingly applied the main DNS using a large-scale domain to reduce the spatial limitation on the laminar–turbulent coexistence.

The numerical conditions of the preliminary and main simulations for η=0.1 are summarized in Table 1 and Table 2, respectively. Long domain sizes of 51.2h and 409.6h were employed in the axial direction to capture a single turbulent puff and expected multiple puffs, whereas the radial and azimuthal domain lengths were of geometrically determined values of *h* and 2π, respectively. The grid resolutions have been confirmed to be fine such that fine-scale eddies in turbulent patches are well resolved, at least for the particularly interesting transitional regime of $Re_{w}\leq 1600$.

Equations (1) and (2) were discretized using a staggered central finite-difference method, where the fourth-order central difference scheme was used in both *x* and θ, along with the second-order scheme in *r* on a non-uniform radial grid. A time advancement was performed using a fractional-step second-order Adams–Bashforth scheme in combination with a Crank–Nicolson scheme for the radial viscous term. The Courant–Friedrichs–Lewy (CFL) condition was continuously monitored in all directions, and accordingly, the time-step Δt constraint for the nonlinear terms was enforced to ensure stability. The details of the numerical method were reported in the literature [34,53]. The code validation carried out is discussed in the next section.

## 3. Preliminary Simulations

**Figure 2:**
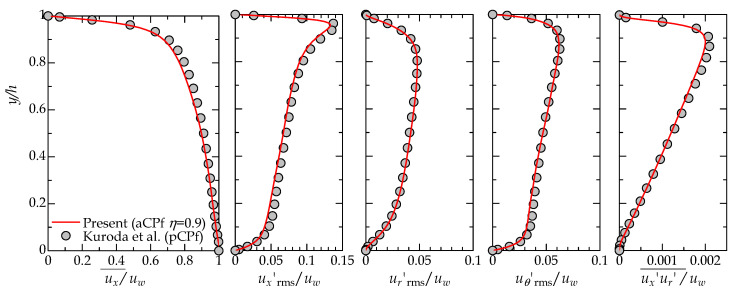
Code validation by comparison with a previous direct numerical simulation (DNS) study on Couette–Poiseuille flow at $Re_{w}=6000$. (Left) Mean streamwise velocity profile; (middle three panels) root-mean-square values of velocity fluctuations in the streamwise, wall-normal (radial), and spanwise (azimuthal) directions; and (right) Reynolds shear stress. Lines and symbols represent the results obtained by this study for the annular Couette–Poiseuille flow (aCPf) with η=0.9, and the result by Kuroda et al. [43] for plane Couette–Poiseuille flow (pCPf), respectively. Here, the wall-normal coordinate *y* represents the distance from the inner (bottom) wall, that is, $y=r-r_{\mathrm{in}}$.

The reliability of the current simulation code may be demonstrated through a comparison with the existing pCPf DNS database at a comparable Reynolds number. Kasagi and coworkers [43,54] applied a DNS of several pCPfs and released their database obtained, from which a condition of $(Re_{w},\left(p\right))=\left(6000,15.96\right)$ was chosen for the code validation during this test. At this Reynolds number and the mean pressure gradient, the pCPf is under a fully turbulent state throughout the channel, and no large-scale intermittency occurs. Its friction Reynolds number $Re_{\tau }$, normalized by the friction velocity on the fixed wall and the half width of the gap, is 154. Kuroda et al. [43] adopted a spectral method with a 128×128 Fourier series in the horizontal directions and Chebyshev polynomials up to order 96 in the wall-normal direction. Their domain size was 2.5πh×h×πh, whereas our counterpart simulation on an aCPf of η=0.9 employed a nearly equal domain size of 8h×h×π/8 (≈3h at the gap center) in (x,r,θ). Figure 2 shows comparisons of the present mean and second-order statistics. An overbar, such as $\bar{u_{x}}\left(r\right)$, denotes an ensemble-averaged quantity with respect to *t*, *x*, and θ, and subscript ‘rms’ indicates a root-mean-square value. The present control parameters of $Re_{w}$ and F(p) for our aCPf are 6000 and 16.0, respectively, and the resulting friction velocity and the friction Reynolds number on the fixed outer cylinder wall are $0.050u_{w}$ and 151. The present results shown in Figure 2 are in reasonable agreement with the reference study, despite the wall curvature of the aCPf. A noticeable difference is detected only near the fixed wall (y/h≈0.9), where the profile of $\bar{u_{x}}$ exhibits a steep gradient, and thus, those of the streamwise turbulent intensity ${u_{x}^{\prime }}_{\mathrm{rms}}$ and Reynolds shear stress $\bar{u_{x}^{\prime }u_{r}^{\prime }}$ have peaks. The rather coarse grid resolution and the low-order spatial discretization (our finite difference code versus the previous spectral code) might affect the accuracy of the present simulation. In addition to the peak values of ${u_{x}^{\prime }}_{\mathrm{rms}}$ and $\bar{u_{x}^{\prime }u_{r}^{\prime }}$, the second-order statistics from the present DNS and those of Kuroda et al. [43] agree well, particularly considering the differences in the flow geometry. The current Fortran code has been employed in different studies for several different boundary conditions [34,35,37,38,55], and thus, no further validation will be shown here.

**Figure 3:**
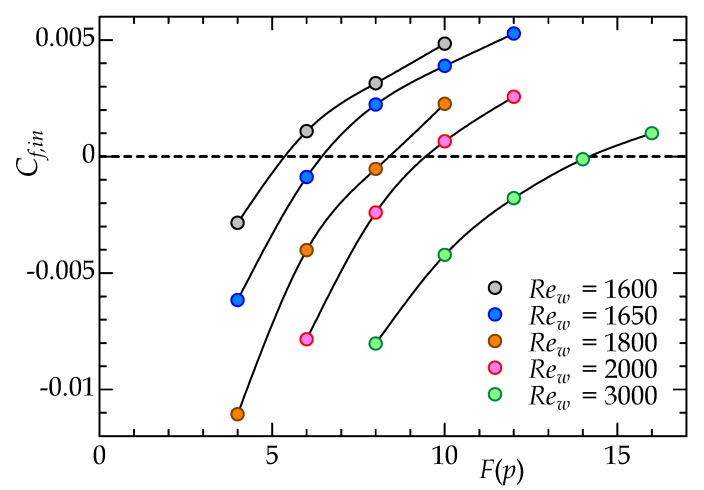
Friction coefficient on the inner cylinder surface as a function of the pressure function F(p) for different Reynolds numbers, obtained through the preliminary DNS study on an aCPf.

In this study, we simulated a low-η annular flow that mimics a CPF by approximating the base flow, or the mean velocity profile, to that in the CPF. In the CPF, the velocity profile reaches its maximum at the pipe center; the velocity gradient becomes zero at the pipe center, and is at maximum on the surface of the pipe (i.e., the outer-cylinder surface). To match the base-flow characteristics of a CPF in an annular system, it is necessary to conduct a parametric investigation on the appropriate magnitude of the pressure gradient applied in the annular channel. As a preliminary analysis, we employed a medium-scale computational domain to reduce the computational cost of the parametric study. The computational domain size is smaller in the *x* direction than the present main analysis shown in Section 4. The streamwise length of the domain, $L_{x}$, was sufficient to capture one turbulent puff. The purpose of the preliminary analysis is to identify the value of the pressure gradient function F(p) at each Reynolds number such that the friction coefficient on the inner cylindrical wall, $C_{f,in}$, is practically zero, where $C_{f,in}$ is defined by the following:

(4) $$C_{f,in}=\frac{\tau_{\mathrm{in}}}{\frac{1}{2}\rho U^{2}},$$

where *U* is the bulk mean velocity obtained through a simulation. The positive/negative sign of $C_{f,in}$ corresponds to the positive/negative velocity gradient on the wall surface of the inner cylinder. Given du/dr<0, $C_{f,in}<0$, and vice versa. Table 1 shows the calculation conditions and the ranges of $Re_{w}$ and F(p) in the preliminary analysis. In the preliminary analysis, the calculation area in the mainstream direction was set to a smaller calculation area than that for the main analysis, but can capture one turbulent puff. Figure 3 shows the F(p) dependence of $C_{f,in}$ at several values of $Re_{w}$ near the global critical value. In each analysis plotted in the figure, a turbulent field with a high Reynolds number at equilibrium for each given F(p) was set as the initial flow field, and the ensemble-averaged $C_{f,in}$ value was acquired after reaching a statistically steady state. Note that laminarization did not occur in any of the cases shown here. In general, as F(p) increases, $C_{f,in}$ increases monotonically while changing from a negative to a positive value. This is consistent with the transition of the mean velocity profile from Couette-like to Poiseuille-like, and it can be confirmed that “the turning point” of F(p) indicating $C_{f,in}=0$ increases with $Re_{w}$. According to this Reynolds-number dependence, an extrapolation predicts a value of F(p) that brings $C_{f,in}=0$ at a lower $Re_{w}$, by which the main DNS analysis in the next section was executed.

## 4. Results and Discussion

The main DNS at $Re_{w}\leq 1600$ for which a laminar–turbulent intermittency was clearly confirmed through the preliminary analysis is presented in this section, and the characteristics of the localized turbulence are discussed. Table 2 summarizes the numerical conditions, including the friction Reynolds number $Re_{\tau }$ that was obtained. As in the preliminary analysis, the radius ratio was η=0.1, and the computational domain was extended only in *x*; however, the grid resolution was not changed. Because $Re_{\tau }$ is lower than that of the preliminary analysis, the grid spacing in terms of the wall units was finer. The table also shows the grid resolutions based on the friction velocity $u_{\tau }$ under each condition:

(5) $$u_{\tau }=\frac{\eta u_{\tau ,in}+u_{\tau ,out}}{\eta +1},$$

where $u_{\tau ,in}$ and $u_{\tau ,out}$ are defined by the corresponding wall shear stress, $\tau_{\mathrm{in}}$, and $\tau_{\mathrm{out}}$, as well as by the relation $\tau =\rho u_{\tau }^{2}$, from which inner units can be defined. For a low Reynolds-number regime of $Re_{w}<1600$, which is of interest in this study, the grid spacings of $\Delta x^{+}<8$, $\Delta r_{\min}^{+}<0.2$, $\Delta r_{\max}^{+}<3$, and $\Delta z^{+}<4$ are comparable to or higher in resolution than those in previous studies [35,37,53]. The initial conditions during each analysis adopted a turbulent field with a one-step-higher Reynolds number, but reduced the Reynolds number adiabatically. In other words, the study was carried out carefully such that the sudden drop in the Reynolds number will not be a proximate cause of laminarization.

### 4.1. Puffs in Annular-Pipe Flow

**Figure 4:**
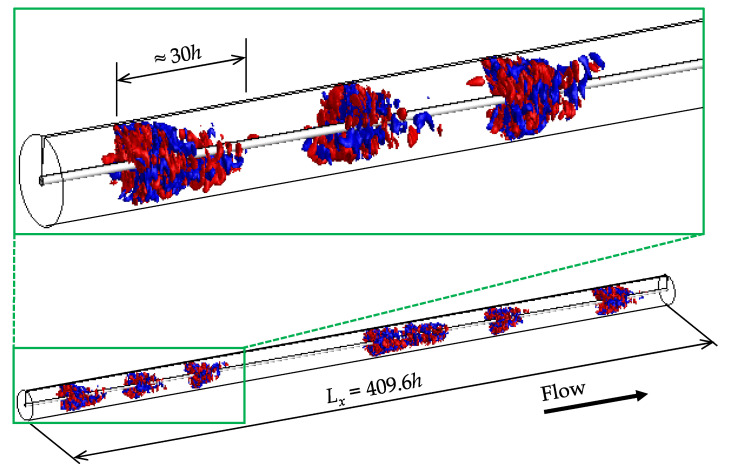
Instantaneous flow field for $Re_{w}=1600$ and F(p)=6.5. Iso-surfaces of radial velocity fluctuation are shown: red, $u_{r}^{\prime }=0.03u_{w}$; blue, $u_{r}^{\prime }=-0.03u_{w}$. The left-to-right direction corresponds to the direction of the main flow, by which the observed puffs propagate. Not to scale.

**Figure 5:**
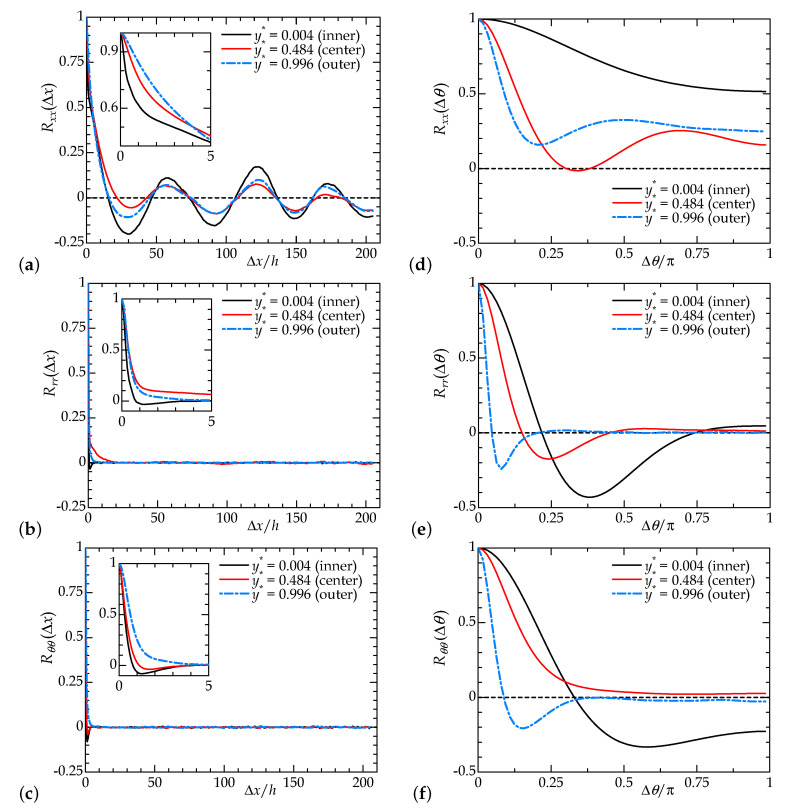
Two-point correlation coefficient of velocity fluctuation for $Re_{w}=1600$ and F(p)=6.5. (**a**–**c**) Streamwise spatial correlation as a function of Δx, and (**d**–**f**) azimuthal correlation as a function of Δθ. (**a**,**d**) Auto-correlation of streamwise velocity component $u_{x}^{\prime }$, (**b**,**e**) that of $u_{r}^{\prime }$, and (**b**,**e**) that of $u_{\theta }^{\prime }$. Here, the reference radial position $r_{\mathrm{ref}}$ is translated as the inner-wall-normal height $y^{*}=r_{\mathrm{ref}}-r/h$.

Figure 4 presents a three-dimensional visualization of localized turbulence in the form of puffs, which is observed as an equilibrium state reached after a lengthy simulation under the condition of $Re_{w}=1600$ and F(p)=6.5. The turbulent region can be clearly detected by showing the radial velocity fluctuations or the wall-normal velocity component. The threshold value of $\pm 0.03u_{w}$ for the iso-surface was arbitrarily chosen to extract its typical arrowhead shape similar to that of a puff. A slight change in this threshold value does not significantly affect the interpretation of the present results. In the snapshot, multiple turbulent patches, called ‘puffs’ hereafter, can be confirmed to be distributed intermittently with respect to the streamwise direction. The blank regions between neighboring puffs can be regarded as being in a laminar flow because of an insignificant fluctuating velocity, implying the well-established coexistence of laminar and turbulent regions in the aCPf. As is clear from the enlarged figure, the puff has an arrowhead shape, and the puff extends downstream in the center of the outer pipe. Although the average velocity gradient on the inner cylindrical wall surface is almost zero, this situation is considered to be due to the similar driving mechanism of the puff of the CPF. For the CPF, Shimizu et al. [11] reported that turbulence in the puff originates from low-speed streaks, as well as from streamwise vortices along the (outer-)pipe wall and across the trailing edge of the puff through the Kelvin–Helmholtz instability, which induces velocity fluctuations that propagate downstream faster than the puff itself in the core region. Such a driving mechanism of the puff is also common to the present aCPf with nearly zero $C_{f,in}$. The streamwise size of each puff is approximately 30 times the gap width *h*, which corresponds to 15 times the hydraulic diameter, and is consistent with that of the puff observed in the CPF [1,7,8,12]. The array of puffs seems variable in intervals, but is likely not less than 30h. The wavelength and periodicity of the puffs are examined using two-point correlation functions of the turbulence quantities. In the *x* and θ directions, the auto-correlation coefficients are defined as follows:

(6) $$R_{ii}\left(\Delta x\right)=\frac{\bar{u_{i}^{\prime }\left(x,r_{\mathrm{ref}},\theta \right)u_{i}^{\prime }\left(x+\Delta x,r_{\mathrm{ref}},\theta \right)}}{{u_{i}^{\prime }}_{\mathrm{rms}}\left(r_{\mathrm{ref}}\right)\cdot {u_{i}^{\prime }}_{\mathrm{rms}}\left(r_{\mathrm{ref}}\right)}$$

and

(7) $$R_{ii}\left(\Delta \theta \right)=\frac{\bar{u_{i}^{\prime }\left(x,r_{\mathrm{ref}},\theta \right)u_{i}^{\prime }\left(x,r_{\mathrm{ref}},\theta +\Delta \theta \right)}}{{u_{i}^{\prime }}_{\mathrm{rms}}\left(r_{\mathrm{ref}}\right)\cdot {u_{i}^{\prime }}_{\mathrm{rms}}\left(r_{\mathrm{ref}}\right)},$$

where i∈(x,r,θ). Figure 5 shows the two-point correlation coefficients of each velocity component for the case visualized in Figure 4. The statistical dataset was accumulated over the time of $5000h/u_{w}$ after achieving a pseudo-equilibrium state of multiple puffs.

From Figure 5a, the axial periodicity and interval of the puff can be estimated. First, we note that the three curves at different $y^{*}$ exhibit consistency, implying that flow state and patterning are only weakly dependent on *y* or *r*. As also plotted in (b) and (c) for the other directional components, fine-scale turbulent structures inside a puff should have a rather short streamwise extent, and indeed, the profiles of $R_{rr}$ and $R_{\theta \theta }$ fall to almost zero at Δx<5h. The profile of $R_{xx}$ also decreases drastically for a small Δx, although its significant oscillation for a long axial extent suggests a spatial coexistence of laminar and turbulent regions rather than turbulent structures, since these two flow states have different mean velocity profiles, particularly near the walls. The oscillations observed in Figure 5a are somewhat strong at both the inner and outer walls, relative to the gap center. The profile of $R_{xx}$ takes the first negative local minimum at Δ≈30h and shows regular spikes at intervals of approximately 60h. The correlation is not zero even at half the computational domain length ($L_{x}/2=204.8h$). Peaks at 60h, 120h, and 180h manifest the presence of seven distinct puffs in $L_{x}$ on average. This suggests that the puffs at this Reynolds number tend to be arranged regularly throughout the axial extent. If the puff spacing is irregular, the correlation coefficient distribution should not show periodic fluctuations and should asymptotically approach zero. This regularity of the puff arrangement may differ from the characteristic of the DP universal class, which should exhibit a wide-scale invariant pattern close to the critical point [39,41]. Mukund and Hof [3] reported a similar aspect on multiple puffs in a CPF, where they referred to the wave-like fashion as ‘puff clustering’; that is, the resultant pattern of clustering puffs was observed to propagate like waves. They also pointed out that interactions between puffs were responsible for the approach to the statistical steady state and strongly affected the percolation threshold. This may predict a difference in the global stability between a single puff (i.e., isolated puffs) and multiple puffs (puff clustering), as discussed in Section 4.2.

The azimuthal two-point correlation functions shown in Figure 5d–f indicate the azimuthal intervals between fine-scale turbulent structures, such as low-speed streaks inside the puff. There exists no large-scale pattern in the azimuthal direction, unlike those of the helically shaped turbulent patches in high-η aPf [35] and aCf [37]. The blue curve in Figure 5d, measured near the outer cylinder wall, only has a peak at Δθ=π/2. The cross-sectional flow pattern observed here consists of four low-speed streaks close to the outer wall spaced at π/2. This azimuthal configuration regarding turbulence inside the puff is in agreement with those found in the CPF [6].

The presence of turbulent equilibrium puffs was observed even at $Re_{w}<1600$, and the flow field finally reached the fully laminar state at $Re_{w}=1500$. Although the space-time diagram (STD) and the turbulent fraction, Ft(t) (the plot of which is shown later), reveal a tendency toward laminarization at $Re_{w}=1525$, one turbulent puff was maintained in the present computational domain at least during the present observation time of >$1.3\times 10^{4}$, and the laminarization was not completed. If normalized by the hydraulic equivalent diameter 2h and bulk velocity *U*, the Reynolds numbers of $Re_{w}=1600$ and 1500 correspond to $Re_{D}=2190$ and 2045, respectively. This range of $Re_{D}=2045$–2190 is close to or slightly narrower than that for the counterpart of the CPF ($Re_{D}=2000$–2700 [1], 2040–2400 [10], 2300–3000 [2], and 2000–2200 [4]). In particular, a discrepancy in the lower bound value of the subcritical transition regime, that is, the global critical Reynolds number, is of interest, although the similarity with the results by Avila et al. [10] is rather surprising. A cause of this discrepancy remains unclear: One of the main causes may be the presence of the inner cylinder, which suppresses turbulent motions across the central axis in the case of an aCPf. Another cause may be the non-slip inner-cylinder surface, which prevents a puff from splitting into two puffs in the case of an aCPf. Shimizu et al. [8] proposed a model process of puff splitting in the CPF, which starts with an azimuthally isolated streak propagating downstream through the laminar–turbulent interface of the puff. An emitted streaky disturbance can be a seed of a “daughter puff,” which spreads again in the azimuthal direction and grows into a turbulent puff after leaving the parent puff sufficiently far away. As a system even closer to the CPF, an ideal aPf with a stress-free boundary condition at the inner wall can be analyzed, although such an unpractical situation will be considered as a future task. In terms of the conjecture that puff splitting is unlikely in the aCPf relative to the CPF, we traced puffs with lengthy simulations, and their STDs are shown in Section 4.2.

### 4.2. Space-Time Diagrams

**Figure 6:**
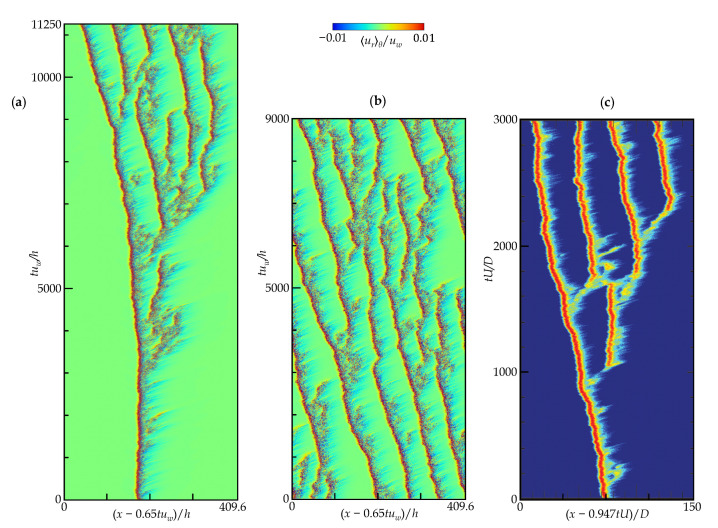
Space-time diagram for (**a**) Case 1 at $Re_{w}=1600$ and F(p)=6.5, (**b**) Case 2 at $Re_{w}=1600$ and F(p)=6.5 with a different initial condition from that in Case 1, and (**c**) a typical discrete expansion process of turbulence in a subcritical transitional pipe flow at Re=2300, cited from Avila et al. [10]. In (**c**), the contour color indicates the cross-sectional average of the streamwise vorticity squared, where red and blue correspond to turbulent puff and laminar regions, respectively, and the Reynolds number Re is based on the mean velocity *U* and the pipe diameter *D*. In (**a**,**b**), the contour shows the azimuthally averaged radial velocity ${\langle u_{r}\rangle }_{\theta }$ at the gap center. The axial distribution is monitored from a moving frame of reference with a speed close to the puff propagation. The temporal development is monitored from t=0, that is, the beginning of each DNS with a higher-$Re_{w}$ field with more puffs; therefore, some initial puffs decayed immediately after the start of the simulation. Not to scale (aspect ratio *x*:*y* = 10:1).

**Figure 7:**
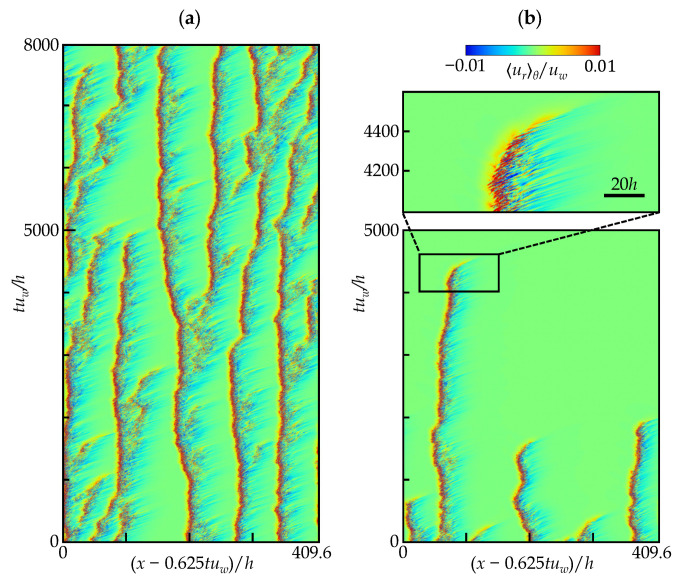
Space-time diagram for (**a**) $Re_{w}=1600$ ($Re_{D}\approx 2190$) and F(p)=6.5, as well as (**b**) $Re_{w}=1500$ and F(p)=5.3 ($Re_{D}\approx 2045$). The contour shows ${\langle u_{r}\rangle }_{\theta }$ at the gap center. The axial distribution was monitored from a moving frame of reference. The same initial condition was applied for all cases presented here. Not to scale (aspect ratio *x*:*y* = 10:1).

**Figure 8:**
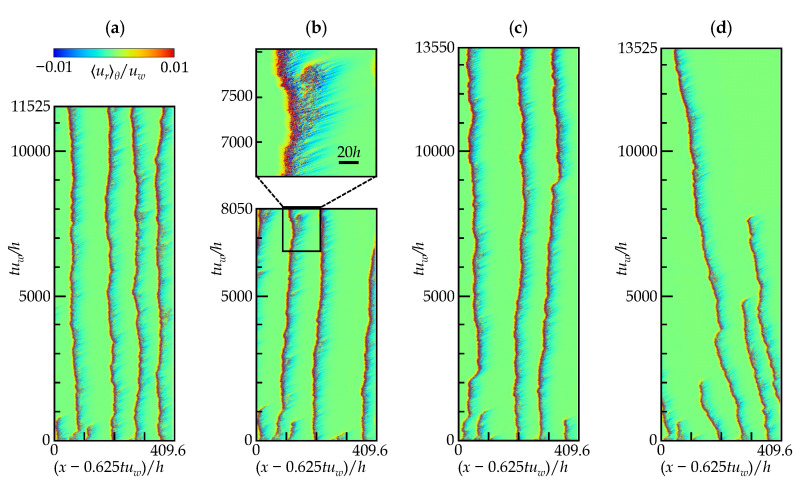
Space-time diagram for (**a**) $Re_{w}=1550$, (**b**) $Re_{w}=1540$, (**c**) $Re_{w}=1530$, and (**d**) $Re_{w}=1525$: see Table 1 for each given F(p) value. The contour shows ${\langle u_{r}\rangle }_{\theta }$ at the gap center. The axial distribution is monitored from a moving frame of reference. The same initial condition was applied for all cases presented here. Not to scale (aspect ratio *x*:*y* = 10:1).

As for the puff turbulence in CPF, it is well known that a turbulent puff can split into two puffs over time, the turbulence between puffs should attenuate and become a laminar pocket, and one or both puff(s) should decay quasi-stochastically because of their finite lifetime [3,6,8,9]. Avila et al. [10] observed the puff-turbulence sustainment only due to puff-splitting events that have time scale shorter than the puff-decay time scale. These features may be identified from the temporal development of the puff spatial distribution. The STDs of the present aCPf are shown in Figure 6, Figure 7 and Figure 8, where the horizontal axis is the streamwise coordinate in a frame of reference moving at a certain velocity, and the vertical axis represents the dimensionless time at each Reynolds number. The frame-moving velocity is nearly the mean gap-center velocity, which also corresponds to the propagation velocity of an observed single puff. The color contour shows the azimuthal average of the radial velocity at mid-gap, ${\langle u_{r}\rangle }_{\theta }=\int_{0}^{2\pi }u_{r}\left(x,h/2,\theta ,t\right)d\theta /2\pi$, such that the laminar and turbulent regions can be clearly distinguished. Although the apparent length of each turbulent puff depends on the criterion used to discriminate it from the surrounding laminar flow, a different choice does not change the qualitative conclusions obtained.

We first present the results for $Re_{w}=1600$ and F(p)=6.5, as discussed in Section 4.1. The flow field visualized in Figure 4 was first achieved through an adiabatic decrease in $Re_{w}$ (with a change in F(p), accordingly) from a fully turbulent regime, and was then used as the initial condition for the following simulation to trace the behavior of the puff in the phase diagram of the $L_{x}$-space and time for as long as possible. The STD obtained is shown in Figure 6b, which monitors the pattern starting from an initial state with several puffs—the isolated turbulent patch featured as a red and blue segment at given time *t*. The overall puff pattern remains intrinsically spatiotemporally intermittent and exhibits both puff decay and splitting very frequently. These individual puffs have statistically well-defined lengths, similar to those in a CPF [8]. The number of puffs captured in the present domain is roughly constant between 5 and 7, and it is again confirmed that the puff intervals tend to be constant even if splitting or attenuation occurs in each individual puff. For this reason, Figure 5a reveals regular oscillations in the correlation function $R_{xx}\left(\Delta x\right)$, while a snapshot visualized in Figure 4 happens to have no periodicity in the puffs when considering the complete pipe length. Figure 6b may invoke an STD obtained from experimental and numerical observations of a DP-like feature in other flow systems [39,42]. Another DNS labeled as Case 1 was repeated for the same parameter set of $\left(Re_{w},F\left(p\right)\right)$, but with a different initial condition with a single puff, which was prepared from a lower-$Re_{w}$ DNS (see Figure 6a). The initial single puff is sustained for a long period of >$5000h/u_{w}$, during which it splits irregularly, the first time at $tu_{w}/h\approx 3000$ and the second time at $tu_{w}/h\approx 4000$, but both newborn puffs decay after they are separated from their parents. A newly emitted daughter puff by the third splitting $tu_{w}/h\approx 5500$ grows and successively produces grandchild puffs. In addition, there are many signs of puff splitting. The puff turbulence eventually covers the entire domain, yet is intrinsically patchy, as in Case 2. It can be concluded that, in an aCPf similar to a CPF, the turbulent puff can split, regardless of the initial field, in qualitative agreement with a typical STD sample of a CPF [10], as displayed in Figure 6c.

Figure 7a shows the STD of $Re_{w}=1600$ and F(p)=6.5 (Case 2), but the speed of the moving frame of reference is modified such that puffs appear to be stationary with respect to space. With this adjustment, the propagation speed of the puff can be estimated as approximately $0.625u_{w}$. According to Figure 6a, when tracking a single puff in Case 1, the propagation speed is slightly faster and ≈$0.65u_{w}$. The result is reasonable because the bulk velocity generally decreases with the expanding turbulent region. Figure 7b is an STD at $Re_{w}=1500$ with the same horizontal coordinate of $(x-0.625u_{w})/h$, showing the eventual return to laminar flow. Once a puff starts to decay, its turbulent patch seems to accelerate slightly and takes approximately $300u_{w}/h$ to attenuate completely. Before that, it took more than $4200h/u_{w}$ before the system settled to the fully laminar state. While the flow at $Re_{D}=2190$ of Figure 7a exhibits frequent puff splitting or those signs during a period of $tu_{w}/h\approx 5000$, the flow at $Re_{D}=2045$ in Figure 7b undergoes only the puff decay with no puff splitting, and the flow field simply reached a laminar flow.

We further investigated the intermediate range between the two above-discussed cases ($2045<Re_{D}<2190$) to elucidate the trends in the frequency or time of the puff-splitting events. Figure 8 presents an STD at each control-parameter set. In all DNSs presented in the figure, the initial conditions are exactly the same. In the figure, six puffs can be seen initially, but two or three of them decay immediately, particularly in the lower-Reynolds-number cases. At the lowest $Re_{w}$ shown in Figure 8d, puffs disappear one after another on a time scale of $O(1000h/u_{w})$, and finally, one puff remains. There is no sign of decay in the surviving puff even after 13,000$h/u_{w}$, but it is likely that the puffs will stochastically disappear and laminarize if a much longer simulation is available. This might also be true for the other cases presented here. At $Re_{w}<1600$, no puff splitting was observed, resulting in only puff damping. In only Figure 8b, a sign of puff splitting is detected at $tu_{w}/h\approx 7500$, although the “daughter puff” is not perfectly formed, and is finally attenuated before leaving the parent puff. Note here that a further DNS indicates no qualitative change in the flow pattern at least until $tu_{w}/h=11,500$ also for $Re_{w}=1540$, although not shown in the figure. According to a similar type of study [10], the puff splitting in the CPF was observed both numerically and experimentally for $Re_{D}>2200$, whereas clear splitting was measured in their experiments down to $Re_{D}=2025<Re_{g}$(=2040). If our observations were continued as long as $10^{7}$ outer time units, as Avila et al. [10] experimentally did, the current system of the aCPf could exhibit a puff-splitting event even below the true $Re_{g}$, which is not exactly determined as of now. At least, it can be said that the puff decay and splitting rates at this stage differ strongly from those observed at $Re_{w}=1600$ ($Re_{D}=2190$). As for this regime, a conclusion similar to an experimental study on a CPF [3] can be drawn, i.e., the cluster of puffs in a wave-like fashion results in fewer puff-splitting events in the STD, whose visual appearance differs from the STD for a DP universality class. Such well-organized distances between active sites (corresponding to the puffs) and the absence of splitting events are different features from those of the DP.

**Figure 9:**
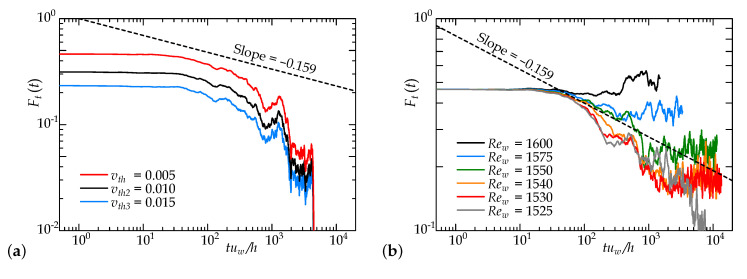
Time series of turbulent fractions: (**a**) for Re=1500 with different threshold values and (**b**) for Re=1525–1600 with a threshold value of $v_{th}$.

Figure 9 shows the temporal change in the turbulent fraction, $F_{t}\left(t\right)$, which is the spatial ratio of the turbulent region to the entire calculated region, including both the turbulent and laminar regions. Here, $F_{t}\left(t\right)\approx 1$ indicates a fully turbulent state, and $F_{t}\left(t\right)=0$ is a fully laminar state. We set a threshold $v_{th}$ to distinguish between laminar and turbulent regions such that $F_{t}\left(t\right)\approx 0.5$ in the Reynolds number region where turbulent puffs densely appear in an axial extent, as in the case of $Re_{w}=1600$ and F(p)=6.5 visualized in Figure 7a. Figure 9a shows the temporal change of $F_{t}\left(t\right)$ at $Re_{w}=1500$ and F(p)=5.3, that is, the case diagnosed as a laminar regime by a visualization in Figure 7b, employing three different threshold values ($v_{th},v_{th2}$, and $v_{th3}$). It can be confirmed that the time change of $F_{t}\left(t\right)$, particularly the gradient of the curve, does not depend on the threshold value. When $v_{th}=0.005$, the temporal changes in $F_{t}\left(t\right)$ at several Reynolds numbers below $Re_{w}=1600$ are plotted in Figure 9b. In the vicinity of the critical point, a (1+1)-D DP universality class should obey a power law of $F_{t}\left(t\right)\propto t^{-0.159}$ over time. From the figure, the current data at $Re_{w}=1575$–1550 seem to be consistent with (1+1)-DP, although more data and more exponents will be needed to properly confirm this trend. However, it should be noted that, for $Re_{w}\leq 1550$, none of the puffs split and turbulent puffs were only attenuated, as shown in Figure 8a. This result suggests that a value close to the critical exponent of DP can be obtained even under a non-DP phenomenon of a simple decaying process without splitting. We should regard this result as a ‘spurious’ DP feature because the puff splitting (or an active site that creates offspring) is a requisite for the critical point and, hence, DP behavior. In other words, this reminds us to take caution regarding the judgment of a DP within the laminar–turbulent intermittency.

## 5. Conclusions

We performed direct numerical simulations (DNSs) of the concentric annular Couette–Poiseuille flow (aCPf) and investigated the laminar–turbulent intermittent field of the so-called puff turbulence, particularly during its subcritical transition. From previous studies, the laminar–turbulent intermittency in annular flows (a pure Couette flow [37] or Poiseuille flow [34]) exhibits the helically shaped turbulent pattern with bi-directional spatial intermittency and puff turbulence with uni-directional intermittency, depending on the radius ratio. This fact leads to a unified understanding of the formation of localized turbulence patterns of different systems, including planar and circular pipe flows (CPFs); however, these analyses were conducted under conditions in which the basic velocity profiles do not qualitatively match those of the CPF. In this study, the radius ratio (of the inner/outer radii) was as low as 0.1, and the mean pressure gradient was imposed such that the inner-cylinder surface had a zero velocity gradient on average, so that the CPF was alternatively simulated by an annular system. Multiple puffs were demonstrated using a long computational domain in the axial direction, and the presence or absence of the puff-splitting event and its onset Reynolds number were investigated using a long-term DNS. The Reynolds number was reduced adiabatically from the fully turbulent field, and the following results were obtained.

- At $Re_{w}=1600$, puff-splitting events occur along with stochastic puff decay, resulting in wave-like fashion of multiple puffs with constant intervals.
- At $Re_{w}<1600$, no puff-splitting event occurs, but initially given individual puffs survive over a present observation time of at least $10^{4}h/u_{w}$, maintaining the intervals among the puffs.
- At $Re_{w}\approx 1550$, a ‘spurious’ feature of (1+1)D-DP was detected during the quenching process even without puff splitting, and a lower $Re_{w}$ deviates from the DP critical exponent.
- At $Re_{w}=1500$, the flow becomes fully laminar after the non-trivial finite lifetime of the puff.
- The range of $Re_{w}=1500$–1600 (with the accordingly changed F(p)) corresponds to the bulk Reynolds-number range of $Re_{D}=2045$–2190 based on the hydraulic diameter and bulk velocity.

The question considered in this study was whether puff splitting can occur in an aCPf, which essentially has a non-slip inner cylinder. In fact, puff splitting was clearly observed at $Re_{w}=1600$, and a sign of splitting was detected at $Re_{w}=1540$, which may be close to the global critical point, $Re_{g}$. This result guarantees that the planar system and the in-pipe system can be linked via the annular system. Near the criticality, oblique turbulent stripes grow or split in the longitudinal direction of the band, but the mainstream directional splitting, as seen in the CPF, is less pronounced in the planar flows. Our results suggest that the localized structures seen in both the planar and pipe flows can cause mainstream directional splitting. However, we should note that no completed puff splitting was detected near $Re_{g}$. The puff splitting could be observed for $Re_{w}<1600$ and even below $Re_{g}$ by increasing both the observation time and domain by orders of magnitude. Such a task to explore the exact $Re_{g}$ value as well as the Reynolds-number dependence of the puff-splitting time scale near $Re_{g}$ is a challenging one that is almost impossible at present. Another possible approach is to study lifetimes of single puffs [56] and time scales of splitting [10] at conditions away from $Re_{g}$, as in earlier studies on the CPF. This may allow us to discuss whether the current system behaves more like quasi-1D Couette flow or like pipe flow, as done by Shi et al. [57] for a pCf. We would like to report on this issue in another paper. Moreover, the characterization of a DP universal class remains skeptical. Similarly to the critical phenomena of the DP universal class, the region of the absorbing state (laminar-flow gap among puffs) should increase as the criticality approaches. From these facts, it is important to verify the DP feature after further expanding the axial computational domain. In addition, since the transition process of an aCPf has a dependence on the radius ratio and F(p), a parametric study will also be addressed in the future.

## Figures and Tables

**Table 1:** Numerical conditions for preliminary DNS with a moderate computational domain.

η	0.1
Domain size	$L_{x}\times L_{r}\times L_{\theta }=51.2h\times h\times 2\pi$
Number of grids	$N_{x}\times N_{r}\times N_{\theta }=512\times 64\times 128$
$Re_{w}$	1600–3000
F(p)	4.0–16.0

**Table 2:** Numerical conditions for the main DNS with a long domain. The grid resolutions of (Δx,Δr,Δθ) are described in their dimensionless form based on $u_{\tau }$ and μ/ρ. The minimum and maximum Δr of the radial direction, in which we used non-uniform grids, are shown. ${}^{†}$ Laminar values from a laminarized case.

η	0.1							
Domain Size	$L_{x}\times L_{r}\times L_{\theta }=409.6h\times h\times 2\pi$							
Number of Grids	$N_{x}\times N_{r}\times N_{\theta }=4096\times 64\times 128$							
$Re_{w}$	3000	1600	1575	1550	1540	1530	1525	1500 ${}^{†}$
$Re_{\tau }\left(=h^{+}\right)$	70.2	36.0	34.1	33.4	33.1	32.7	32.4	31.5
F(p)	14.2	6.5	6.0	5.8	5.7	5.6	5.5	5.3
$\Delta x^{+}$	14.1	7.14	6.82	6.68	6.61	6.53	6.47	6.30
$\Delta r_{\min}^{+}$	0.37	0.19	0.18	0.18	0.17	0.17	0.17	0.17
$\Delta r_{\max}^{+}$	4.44	2.26	2.16	2.11	2.09	2.07	2.05	1.99
$r_{\mathrm{in}}^{+}\Delta \theta$	0.77	0.39	0.37	0.36	0.36	0.36	0.35	0.34
$r_{\mathrm{out}}^{+}\Delta \theta$	7.66	3.89	3.72	3.64	3.61	3.56	3.53	3.44

## Abbreviations

The following abbreviations are used in this manuscript:

aCf	annular Couette flow
aCPf	annular Couette–Poiseuille flow
aPf	annular Poiseuille flow
CPF	Circular pipe flow
DNS	direct numerical simulation
DP	direct percolation
pCPf	plane Couette–Poiseuille flow
pPf	plane Poiseuille flow
rms	root-mean-square value

## References

[1] Wygnanski I.J., Champagne F.H. (1973). On transition in a pipe. Part 1. The origin of puffs and slugs and the flow in a turbulent slug. J. Fluid Mech..

[2] Nishi M., Ünsal B., Durst F. (2008). Laminar-to-turbulent transition of pipe flows through puffs and slugs. J. Fluid Mech..

[3] Mukund V., Hof B. (2018). The critical point of the transition to turbulence in pipe flow. J. Fluid Mech..

[4] Priymak V. (2018). Direct numerical simulation of quasi-equilibrium turbulent puffs in pipe flow. Phys. Fluids.

[5] Avila M., Mellibovsky F., Roland N., Hof B. (2013). Streamwise-localized solutions at the onset of turbulence in pipe flow. Phys. Rev. Lett..

[6] Hof B., Van Doorne C.W., Westerweel J., Nieuwstadt F.T., Faisst H., Eckhardt B., Wedin H., Kerswell R.R., Waleffe F. (2004). Experimental observation of nonlinear traveling waves in turbulent pipe flow. Science.

[7] Moxey D., Barkley D. (2010). Distinct large-scale turbulent-laminar states in transitional pipe flow. Proc. Natl. Acad. Sci. USA.

[8] Shimizu M., Manneville P., Duguet Y., Kawahara G. (2014). Splitting of a turbulent puff in pipe flow. Fluid Dyn. Res..

[9] Barkley D. (2016). Theoretical perspective on the route to turbulence in a pipe. J. Fluid Mech..

[10] Avila K., Moxey D., de Lozar A., Avila M., Barkley D., Hof B. (2011). The onset of turbulence in pipe flow. Science.

[11] Shimizu M., Kida S. (2009). A driving mechanism of a turbulent puff in pipe flow. Fluid Dyn. Res..

[12] Barkley D., Song B., Mukund V., Lemoult G., Avila M., Hof B. (2015). The rise of fully turbulent flow. Nature.

[13] Coles D. (1965). Transition in circular Couette flow. J. Fluid Mech..

[14] Prigent A., Grégoire G., Chaté H., Dauchot O., van Saarloos W. (2002). Large-scale finite-wavelength modulation within turbulent shear flows. Phys. Rev. Lett..

[15] Barkley D., Tuckerman L.S. (2005). Computational study of turbulent laminar patterns in Couette flow. Phys. Rev. Lett..

[16] Duguet Y., Schlatter P., Henningson D.S. (2010). Formation of turbulent patterns near the onset of transition in plane Couette flow. J. Fluid Mech..

[17] Couliou M., Monchaux R. (2015). Large-scale flows in transitional plane Couette flow: A key ingredient of the spot growth mechanism. Phys. Fluids.

[18] De Souza D., Bergier T., Monchaux R. (2020). Transient states in plane Couette flow. J. Fluid Mech..

[19] Tsukahara T., Seki Y., Kawamura H., Tochio D. (2005). DNS of Turbulent Channel Flow at Very Low Reynolds Numbers.

[20] Hashimoto S., Hasobe A., Tsukahara T., Kawaguchi Y., Kawamura H. (2009). An Experimental Study on Turbulent-Stripe Structure in Transitional Channel Flow.

[21] Xiong X., Tao J., Chen S., Brandt L. (2015). Turbulent bands in plane-Poiseuille flow at moderate Reynolds numbers. Phys. Fluids.

[22] Tsukahara T., Tillmark N., Alfredsson P. (2010). Flow regimes in a plane Couette flow with system rotation. J. Fluid Mech..

[23] Brethouwer G., Duguet Y., Schlatter P. (2012). Turbulent-laminar coexistence in wall flows with Coriolis, buoyancy or Lorentz forces. J. Fluid Mech..

[24] Reetz F., Kreilos T., Schneider T.M. (2019). Exact invariant solution reveals the origin of self-organized oblique turbulent-laminar stripes. Nat. Commun..

[25] Paranjape C.S., Duguet Y., Hof B. (2020). Oblique stripe solutions of channel flow. J. Fluid Mech..

[26] Shimizu M., Manneville P. (2019). Bifurcations to turbulence in transitional channel flow. Phys. Rev. Fluids.

[27] Tsukahara T., Ishida T. (2014). Lower bound of sub-critical transition in plane Poiseuille flow. Nagare.

[28] Takeda K., Tsukahara T. Subcritical transition of plane Poiseuille flow as (2+1)d and (1+1)d DP universality classes. Proceedings of the 8th Symposium on Bifurcations and Instabilities in Fluid Dynamics (BIFD2019).

[29] Xiao X., Song B. (2020). Kinematics and dynamics of turbulent bands at low Reynolds numbers in channel flow. Entropy.

[30] Kashyap P.V., Duguet Y., Dauchot O. (2020). Flow statistics in the transitional regime of plane channel flow. Entropy.

[31] Manneville P. (2016). Transition to turbulence in wall-bounded flows: Where do we stand?. Mech. Eng. Rev..

[32] Manneville P. (2017). Laminar-turbulent patterning in transitional flows. Entropy.

[33] Tuckerman L.S., Chantry M., Barkley D. (2020). Patterns in wall-bounded shear flows. Annu. Rev. Fluid Mech..

[34] Ishida T., Duguet Y., Tsukahara T. (2016). Transitional structures in annular Poiseuille flow depending on radius ratio. J. Fluid Mech..

[35] Ishida T., Duguet Y., Tsukahara T. (2017). Turbulent bifurcations in intermittent shear flows: From puffs to oblique stripes. Phys. Rev. Fluids.

[36] Ishida T., Tsukahara T. (2016). Friction factor of annular Poiseuille flow in a transitional regime. Adv. Mech. Eng..

[37] Kunii K., Ishida T., Duguet Y., Tsukahara T. (2019). Laminar–turbulent coexistence in annular Couette flow. J. Fluid Mech..

[38] Takeda K., Duguet Y., Tsukahara T. (2020). Intermittency and critical scaling in annular Couette flow. Entropy.

[39] Lemoult G., Shi L., Avila K., Jalikop S.V., Avila M., Hof B. (2016). Directed percolation phase transition to sustained turbulence in Couette flow. Nat. Phys..

[40] Sano M., Tamai K. (2016). A universal transition to turbulence in channel flow. Nat. Phys..

[41] Chantry M., Tuckerman L.S., Barkley D. (2017). Universal continuous transition to turbulence in a planar shear flow. J. Fluid Mech..

[42] Hiruta Y., Toh S. (2020). Subcritical laminar–turbulent transition as nonequilibrium phase transition in two-dimensional Kolmogorov flow. J. Phys. Soc. Jpn..

[43] Kuroda A., Kasagi N., Hirata M. (1995). Direct numerical simulation of turbulent plane Couette-Poiseuille flows: Effect of mean shear rate on the near-wall turbulence structures. Turbulent Shear Flows 9.

[44] Pirozzoli S., Bernardini M., Orlandi P. (2011). Large-scale motions and inner/outer layer interactions in turbulent Couette–Poiseuille flows. J. Fluid Mech..

[45] Orlandi P., Bernardini M., Pirozzoli S. (2015). Poiseuille and Couette flows in the transitional and fully turbulent regime. J. Fluid Mech..

[46] Kun Y., Lihao Z., Andersson H.I. (2017). Turbulent Couette-Poiseuille flow with zero wall shear. Int. J. Heat Fluid Flow.

[47] Nakabayashi K., Kitoh O., Katoh Y. (2004). Similarity laws of velocity profiles and turbulence characteristics of Couette–Poiseuille turbulent flows. J. Fluid Mech..

[48] Klotz L., Wesfreid J.E. (2017). Experiments on transient growth of turbulent spots. J. Fluid Mech..

[49] Klotz L., Lemoult G., Frontczak I., Tuckerman L.S., Wesfreid J.E. (2017). Couette-Poiseuille flow experiment with zero mean advection velocity: Subcritical transition to turbulence. Phys. Rev. Fluids.

[50] Liu T., Semin B., Klotz L., Godoy-Diana R., Wesfreid J.E., Mullin T. (2020). Anisotropic decay of turbulence in plane Couette–Poiseuille flow. arXiv.

[51] Wong A.W., Walton A.G. (2012). Axisymmetric travelling waves in annular Couette–Poiseuille flow. Q. J. Mech. Appl. Math..

[52] Kumar R., Walton A. (2020). Self-sustaining critical layer/shear layer interaction in annular Poiseuille–Couette flow at high Reynolds number. Proc. R. Soc..

[53] Abe H., Kawamura H., Matsuo Y. (2001). Direct numerical simulation of a fully developed turbulent channel flow with respect to the Reynolds number dependence. J. Fluids Eng..

[54] Turbulence and Heat Transfer Laboratory (THTLAB; Nobuhide Kasagi Lab.).

[55] Fukuda T., Tsukahara T. (2020). Heat transfer of transitional regime with helical turbulence in annular flow. Int. J. Heat Fluid Flow.

[56] Avila M., Willis A.P., Hof B. (2010). On the transient nature of localized pipe flow turbulence. J. Fluid Mech..

[57] Shi L., Avila M., Hof B. (2013). Scale invariance at the onset of turbulence in Couette flow. Phys. Rev. Lett..

